# Type I Cryoglobulinemia Associated With Multiple Myeloma: A Case Report

**DOI:** 10.7759/cureus.108183

**Published:** 2026-05-03

**Authors:** Inasse Mourabiti, Yasmine El Ouafa, Monsif FADI, Nouama Bouanani

**Affiliations:** 1 Hematology, Mohammed VI University of Health and Sciences (UM6SS), Casablanca, MAR; 2 Center for Doctoral Studies (CeDoc), Mohammed VI University of Health and Sciences (UM6SS), Casablanca, MAR; 3 Interdisciplinary Laboratory of Biotechnology and Health, Mohammed VI University of Health and Sciences (UM6SS), Casablanca, MAR; 4 Oncopathology, Cancer Biology, and Environment Laboratory, Mohammed VI Faculty of Medicine, Center for Doctoral Studies (CeDoc), Mohammed VI University of Health and Sciences (UM6SS), Casablanca, MAR

**Keywords:** cutaneous vasculitis, hyper-viscosity syndrome, membranoproliferative glomerulonephritis (mpgn), multiple myeloma, type i monoclonal cryoglobulinemia

## Abstract

Cryoglobulinemia (CG) is a rare disease characterized by the presence of circulating immunoglobulins that precipitate at low temperatures and may lead to systemic manifestations.

We report the case of a 73-year-old woman who met the diagnostic criteria for type I cryoglobulinemic vasculitis, presenting with ulceronecrotic, partly purpuric lesions. Skin biopsy revealed vasculitis, and laboratory evaluation revealed an IgG kappa monoclonal gammopathy, leading to multiple myeloma (MM) as the underlying etiology. Clone-directed therapy resulted in marked clinical improvement, with significant regression of cutaneous lesions within two months.

Given its rarity and potential for severe complications, this case highlights the importance of systematically investigating an underlying monoclonal gammopathy in patients presenting with type I cryoglobulinemic vasculitis.

## Introduction

Cryoglobulinemia (CG) refers to the presence of circulating immunoglobulins that precipitate at low temperatures and redissolve upon warming. It is broadly divided into two categories: type I CG, which occurs exclusively in the setting of clonal hematologic proliferations, and mixed CG (types II and III), which is more frequently related to chronic hepatitis C virus infection or autoimmune connective tissue diseases [1].

Hematologic disorders most often linked to type I CG include monoclonal gammopathy of clinical significance, Waldenström macroglobulinemia, non-Hodgkin lymphoma, multiple myeloma (MM), and chronic lymphocytic leukemia. Although type I CG represents only about 10%-15% of all cryoglobulinemic syndromes, it can produce a wide clinical spectrum, ranging from mild symptoms such as purpura and arthralgia to severe, organ-threatening complications including extensive skin necrosis, renal injury, and neurological involvement [2]. These severe forms carry substantial morbidity and mortality.

Previous cohort studies have shown that while approximately three-quarters of patients with type I CG respond to first-line therapy, up to half experience relapse during long-term follow-up [3]. Current management strategies focus both on symptomatic relief and on eradication or control of the underlying clonal B- or plasma-cell disorder, most often of IgM or IgG isotype [4]. The rarity of the disease, its heterogeneous clinical presentation, and the diversity of associated hematologic malignancies have limited the feasibility of large prospective trials. To date, only small series have addressed prognostic factors in type I CG [3].

We describe the case of a 73-year-old woman with MM and type I CG, presenting with necrotic skin lesions and membranoproliferative glomerulonephritis.

## Case presentation

A 73-year-old woman with a medical history of hypertension presented with recurrent ulcerative skin lesions. Her symptoms began in 2017 with small, mildly painful ulcerative lesions on the leg and foot that later evolved into necrosis. She was evaluated by an angiologist and treated with colchicine and acetylsalicylic acid. Seven years later, she experienced a recurrence marked by diffuse, intensely painful ulceronecrotic lesions on both lower limbs. She also reported progressive weakness, headache, and bone pain over the preceding week.

**Figure 1:**
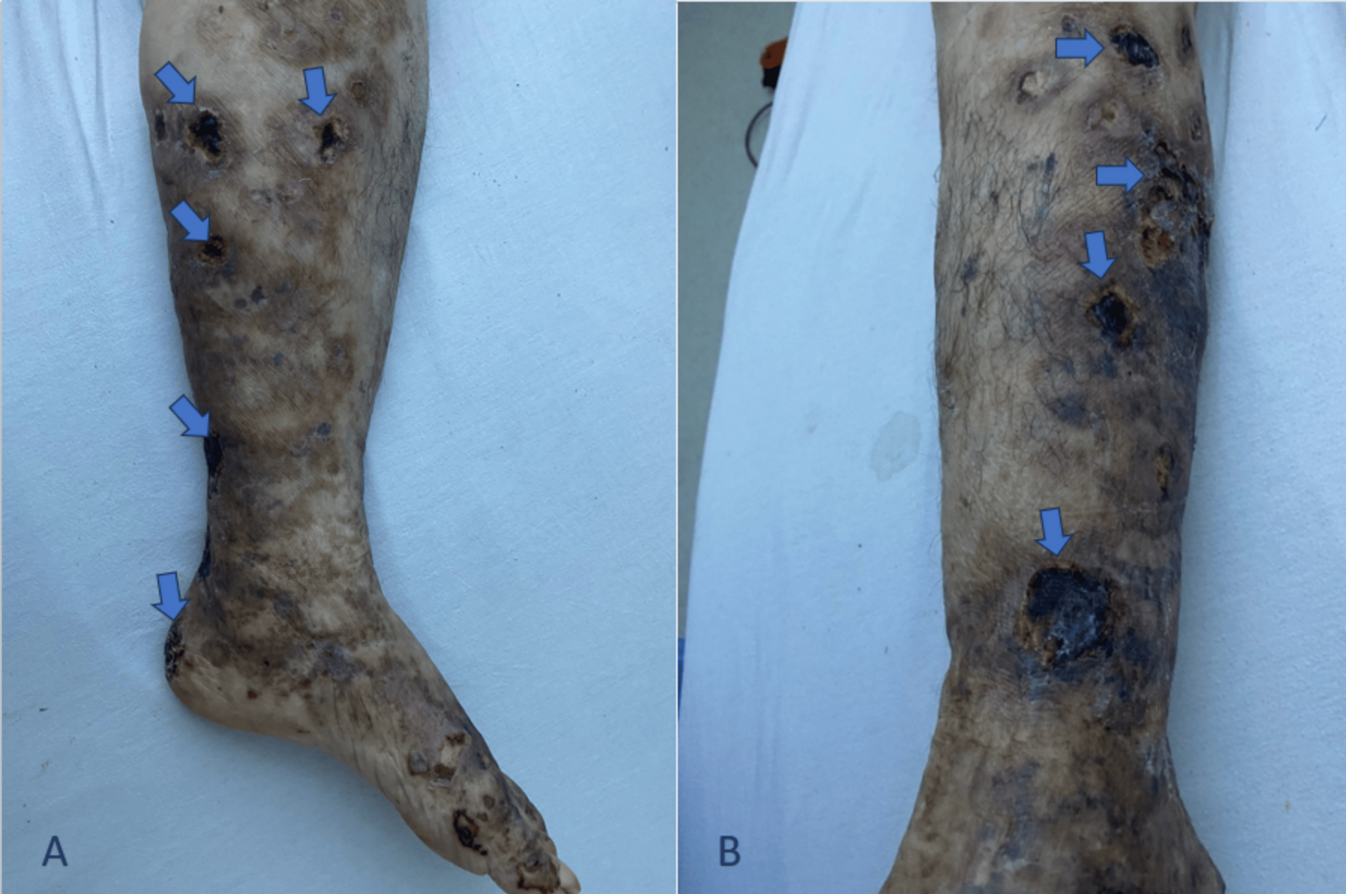
(A-B) Right lower limb showing ulceronecrotic lesions (arrows) with irregular borders, associated with livedo and diffuse hyperpigmentation, consistent with type I cryoglobulinemia.

Physical examination showed ulceronecrotic, partly purpuric lesions on both lower limbs and the dorsal surface of the left foot, with an additional necrotic lesion on the elbow. The background skin appeared atrophic, hyperpigmented, and cicatricial, with areas of ulceration and necrosis measuring approximately 25 mm in diameter (Figure 1).

A skin biopsy revealed findings consistent with vasculitis. Key laboratory findings are summarized in Table 1.

**Table 1:** Laboratory findings at presentation showing anemia, hypoproteinemia, significant proteinuria, and altered renal function, with normal corrected calcium levels.

Parameter	Result	Reference Range
Hemoglobin	8.7 g/dL (↓)	11.8-15 g/dL
Leukocytes	11,950/mm³ (↑)	4,000 - 10,000/mm³
Platelets	300,000/mm³ (N)	150,000 - 400,000/mm³
Total Protein	58 g/L (↓)	64 - 83 g/L
Albumin	37.6 g/L (↓)	40.2 - 47.6 g/L
Urea	1.5 g/L (↑)	0.17 - 0.49 g/L
Serum Creatinine	34.74 mg/L (↑)	5 - 12 mg/L
Calcium	95.77 mg/L (N)	90 - 102 mg/L
Lactate dehydrogenase (LDH)	211 UI/L (N)	80 - 230 UI/L
Beta-2-microglobulin	5 mg/L (↑)	<1.9
24-hour urine protein	2.3 g/24 h (↑)	<0.3 g/24 h
Cryoglobulins	0.556 g/L (↑)	0
Serum-free light-chain kappa	95.15 mg/L (↑)	2.37 - 20.73 mg/L
Serum-free light-chain lambda	9.69 mg/L (N)	4.23 - 27.69 mg/L
Kappa/lambda ratio	9.82 (↑)	0.22 - 1.74

**Figure 2:**
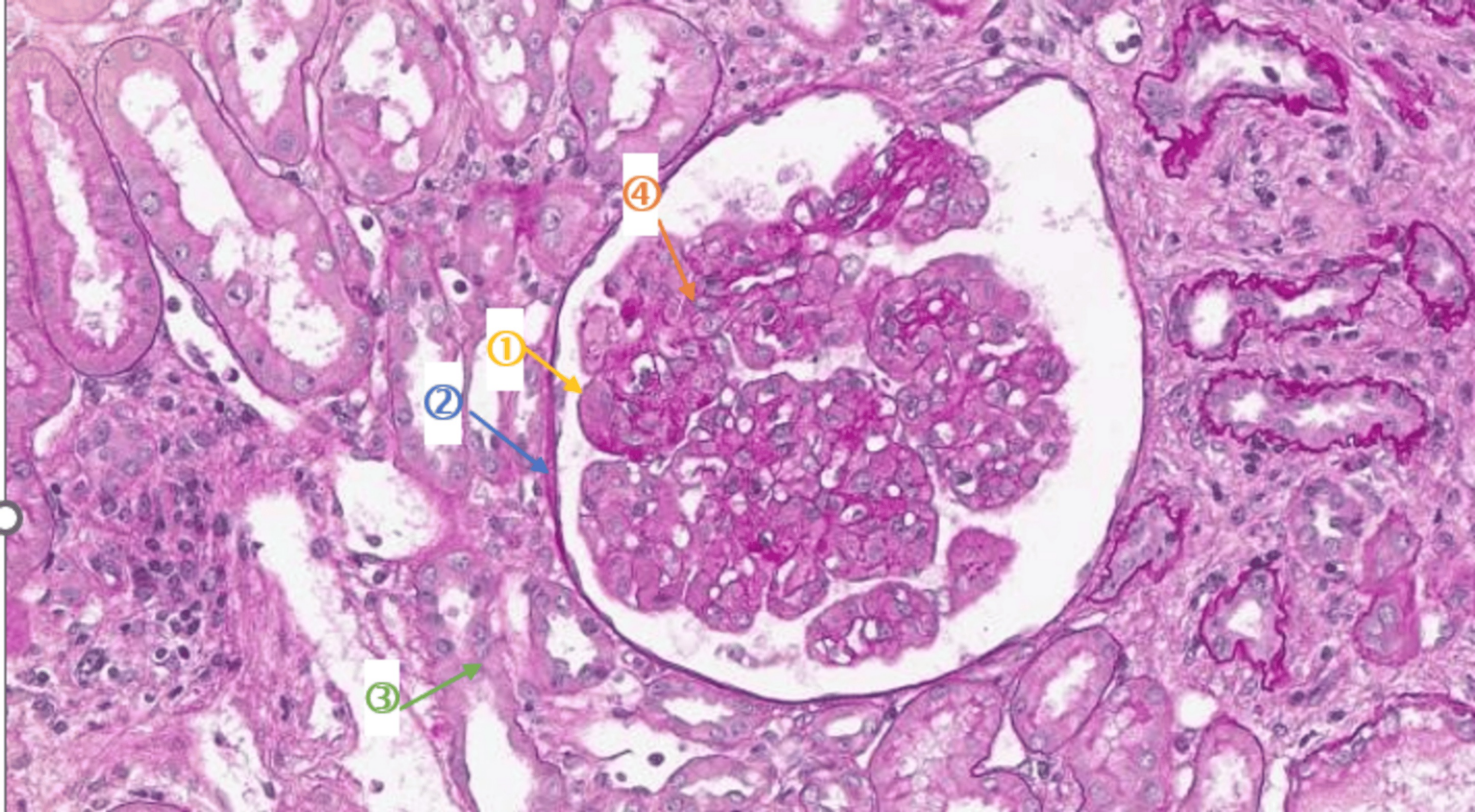
Renal biopsy (HES, ×100) showing membranoproliferative glomerulonephritis with (1) diffuse endocapillary proliferation, (2) mesangial hypercellularity, (3) double-contour formation, and (4) intraluminal hyaline thrombi. These findings are consistent with cryoglobulinemic glomerulonephritis and reflect glomerular injury related to monoclonal immunoglobulin deposition. HES: Hematoxylin-Eosin-Saffron

Given the presence of significant proteinuria, a renal biopsy was performed. Histology showed membranoproliferative glomerulonephritis with diffuse endocapillary proliferation, mesangial hypercellularity, and double-contour formation (Figure 2). Occasional thrombi were present, with 15% interstitial fibrosis and mild fibrous endarteritis. Congo red staining was negative.

Immunofluorescence revealed segmental endomembranous IgG and kappa light-chain deposits without significant IgM, IgA, C3, C1q, or lambda staining. These findings were consistent with CG. Hepatitis C serology was negative, ruling out infection-associated mixed CG.

Serum protein electrophoresis showed marked hypogammaglobulinemia, while immunofixation identified a monoclonal IgG kappa component.

**Figure 3:**
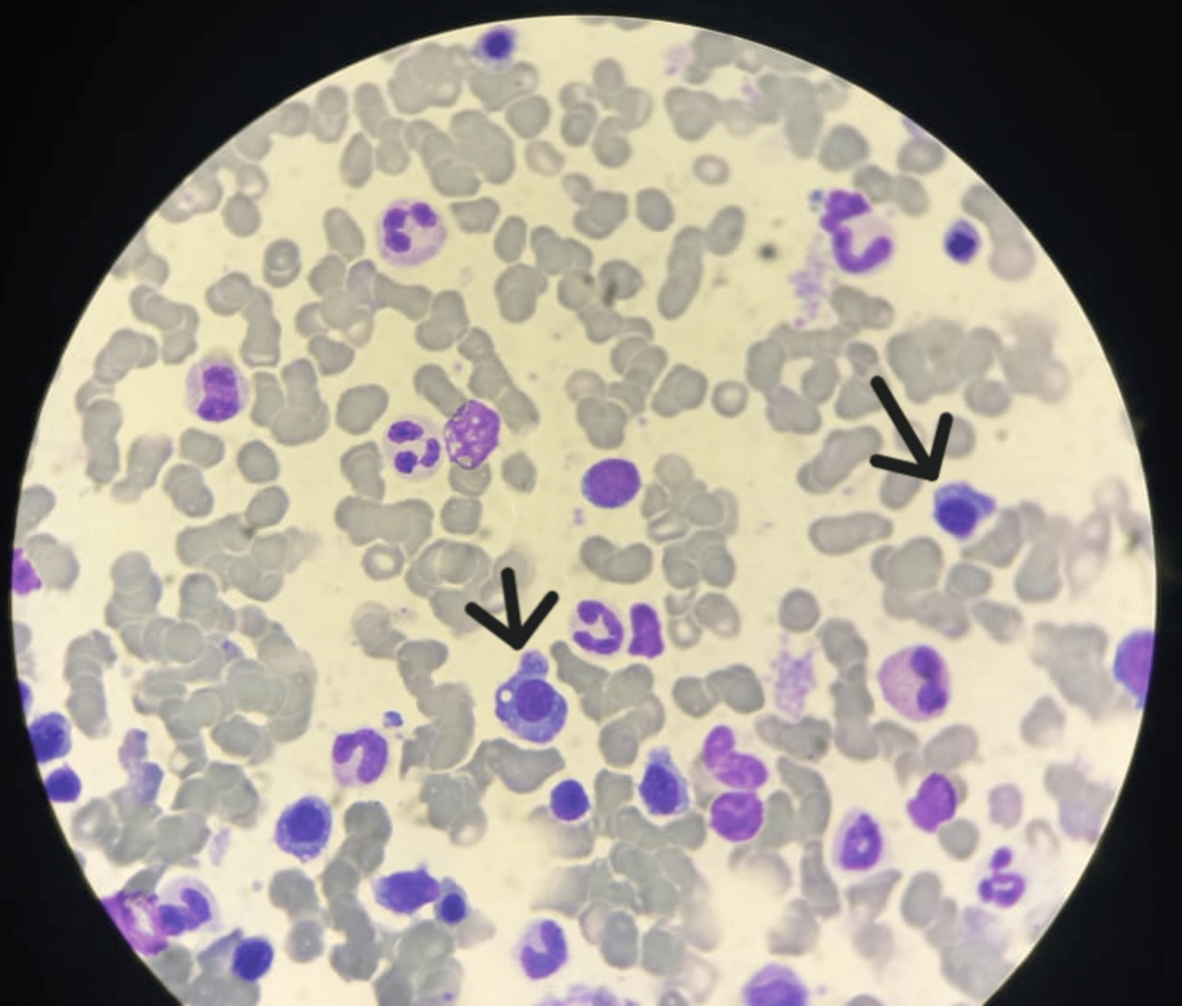
Bone marrow aspiration showing plasma cells with dysplastic morphology (arrows; MGG, ×100). MGG: May-Grünwald-Giemsa

Bone marrow aspiration was hypercellular, showing numerous megakaryocytes, some with dysplastic features. All hematopoietic lineages were represented, with no excess blasts. Plasma cells accounted for 20%, frequently with dysplastic morphology (Figure 3). Cytogenetic analysis showed no chromosomal abnormalities, but fluorescence in situ hybridization (FISH) demonstrated trisomy of the p53 locus at 17p13 in 34% of CD138+ plasma cells.

The final diagnosis was MM IgG kappa R-ISS 3, associated with type I IgG kappa CG.

**Figure 4:**
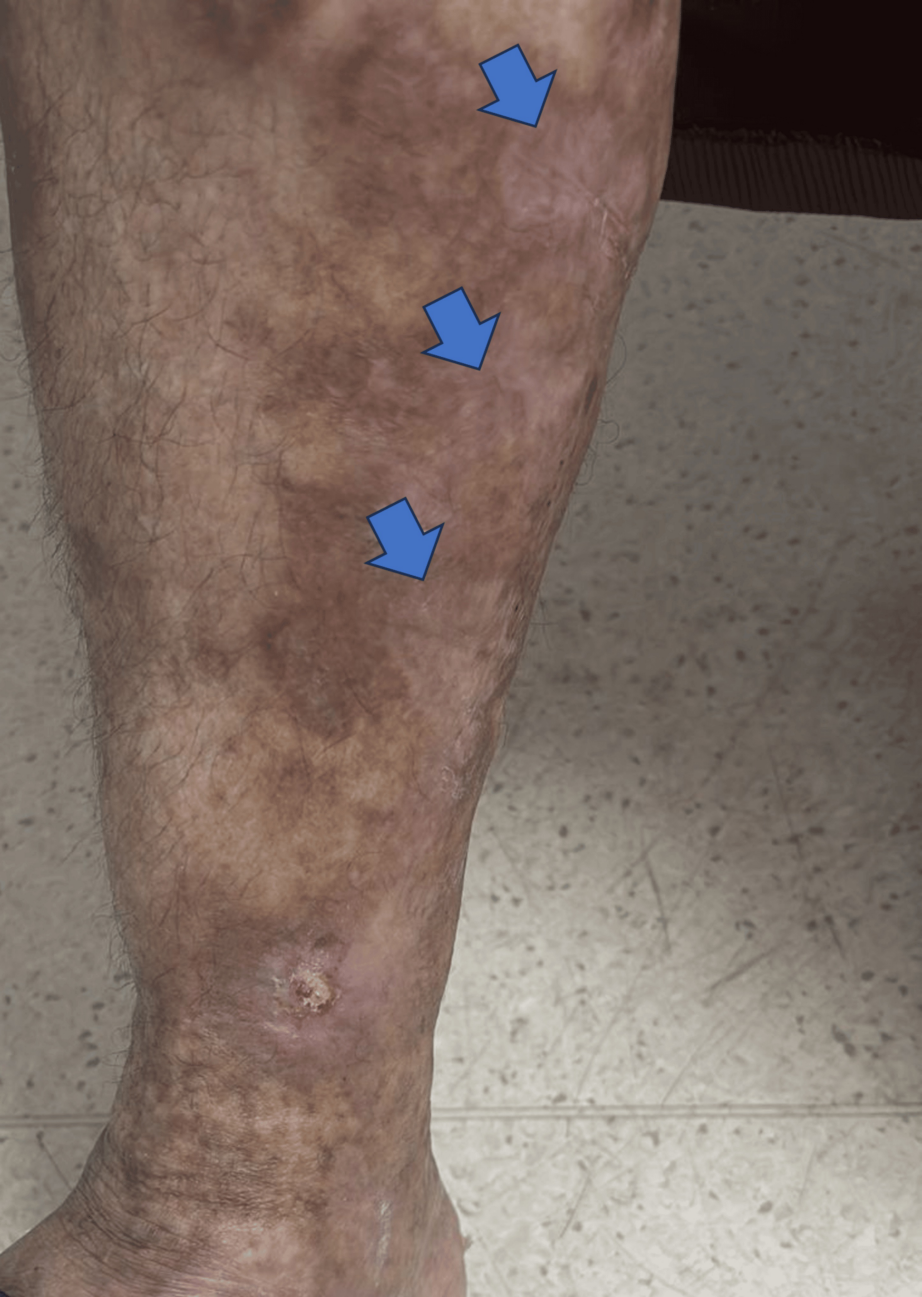
Evolution of lower limb lesions (arrows) showing marked regression of necrotic ulcers and beginning of re-epithelialization after two cycles of VCD. VCD: Bortezomib, Cyclophosphamide, Dexamethasone

**Figure 5:**
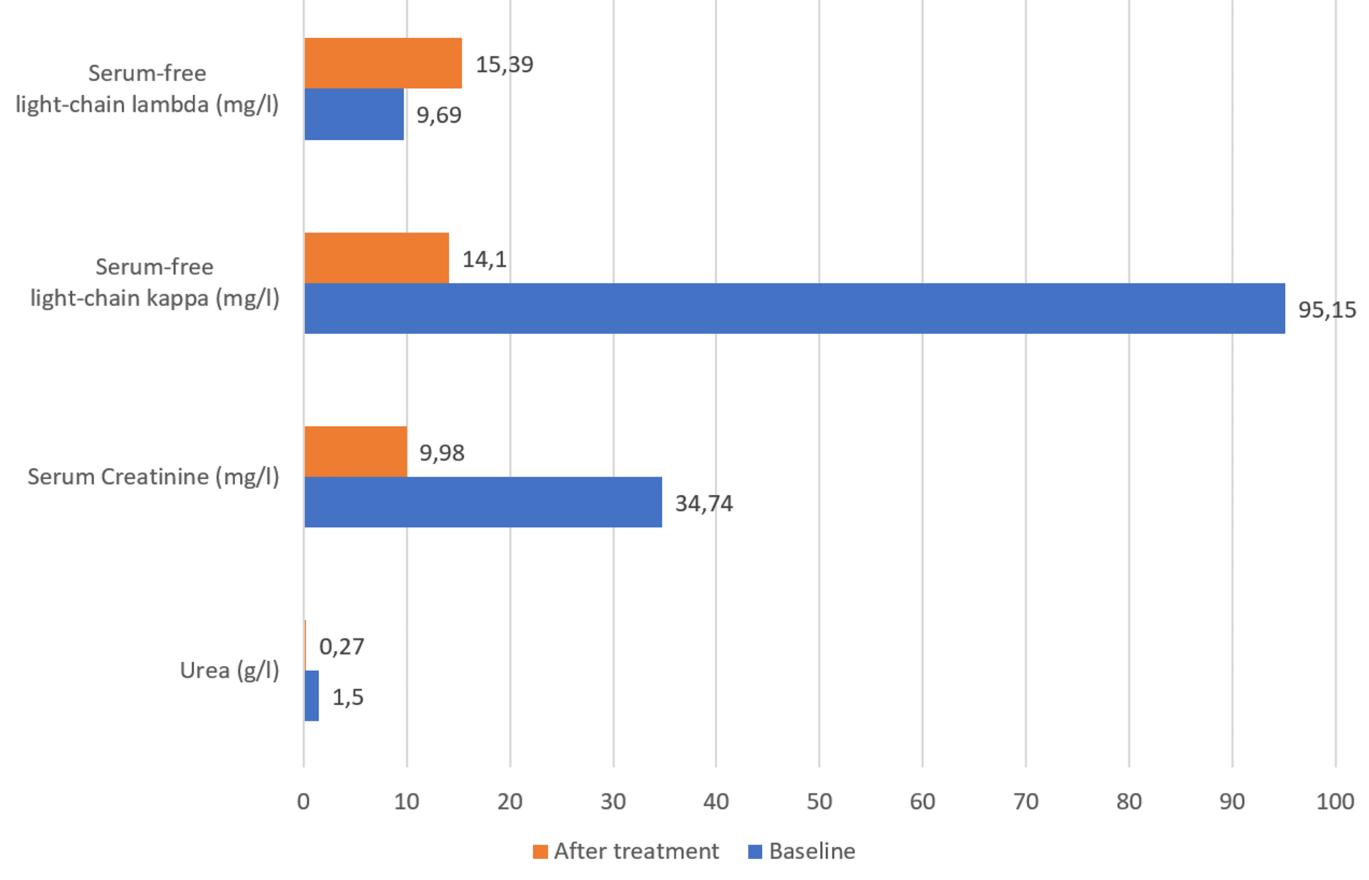
Parameter evolution before and after two cycles of VCD therapy. VCD: Bortezomib, Cyclophosphamide, Dexamethasone

The patient received the VCD regimen (bortezomib 1.3 mg/m², cyclophosphamide 300 mg/m², and dexamethasone 20 mg). Pain improvement was noted after the first cycle. After two cycles, the necrotic ulcers had begun to regress (Figure 4), and cutaneous healing was nearly complete. No new ischemic episodes occurred, and the patient regained partial functional autonomy. Also, parameter evolution before and after two cycles of VCD therapy showed significant improvement (Figure 5).

## Discussion

Our patient illustrates the rare but clinically significant association between MM and type I CG, based on the presence of monoclonal immunoglobulins and compatible clinical and histopathological findings. Although type I CG represents a minority of cryoglobulinemic syndromes, and hematologic malignancies account for <10% of underlying causes, monoclonal plasma-cell disorders remain an important etiology [5].

In published cohorts, MM has been reported in fewer than 10% of type I CG cases, yet case reports, including ours, demonstrate that MM can manifest with severe, organ-threatening cryoglobulinemic vasculitis [3,6].

Cutaneous involvement was the main clinical manifestation in our patient, with recurrent ulceronecrotic lesions complicated by ischemic necrosis. This mirrors epidemiologic data from a Chinese cohort, in which skin manifestations were observed in 57.8% of patients with type I CG [3]. In MM-associated cases, acral ischemia, necrotic ulcers, and oropharyngeal lesions have been described, reflecting the pathogenic role of circulating monoclonal immunoglobulins in vascular occlusion and tissue necrosis [6]. 

Renal involvement represented a second major axis of disease. Our patient had nephrotic-range proteinuria, and the renal biopsy confirmed cryoglobulinemic membranoproliferative glomerulonephritis. This is consistent with reports that monoclonal immunoglobulins in MM or monoclonal gammopathy of renal significance may deposit in glomeruli, producing a heterogeneous spectrum of lesions, including CG-associated forms [7]. Importantly, the biopsy helped differentiate this lesion from myeloma-related cast nephropathy or amyloidosis, both of which were excluded.

Laboratory testing, including cryoglobulin detection, immunofixation, and free light-chain quantification, guided diagnosis. These investigations are central to evaluating suspected CG in patients with plasma-cell disorders [8].

FISH analysis revealed trisomy of the 17p13 locus in plasma cells, supporting the clonal nature of the disease. Unlike 17p deletion (TP53), which is associated with high-risk MM, the prognostic significance of this abnormality remains uncertain. It was not classified as high-risk, and some evidence suggests it may ameliorate the impact of other high-risk markers [9].

**Figure 6:**
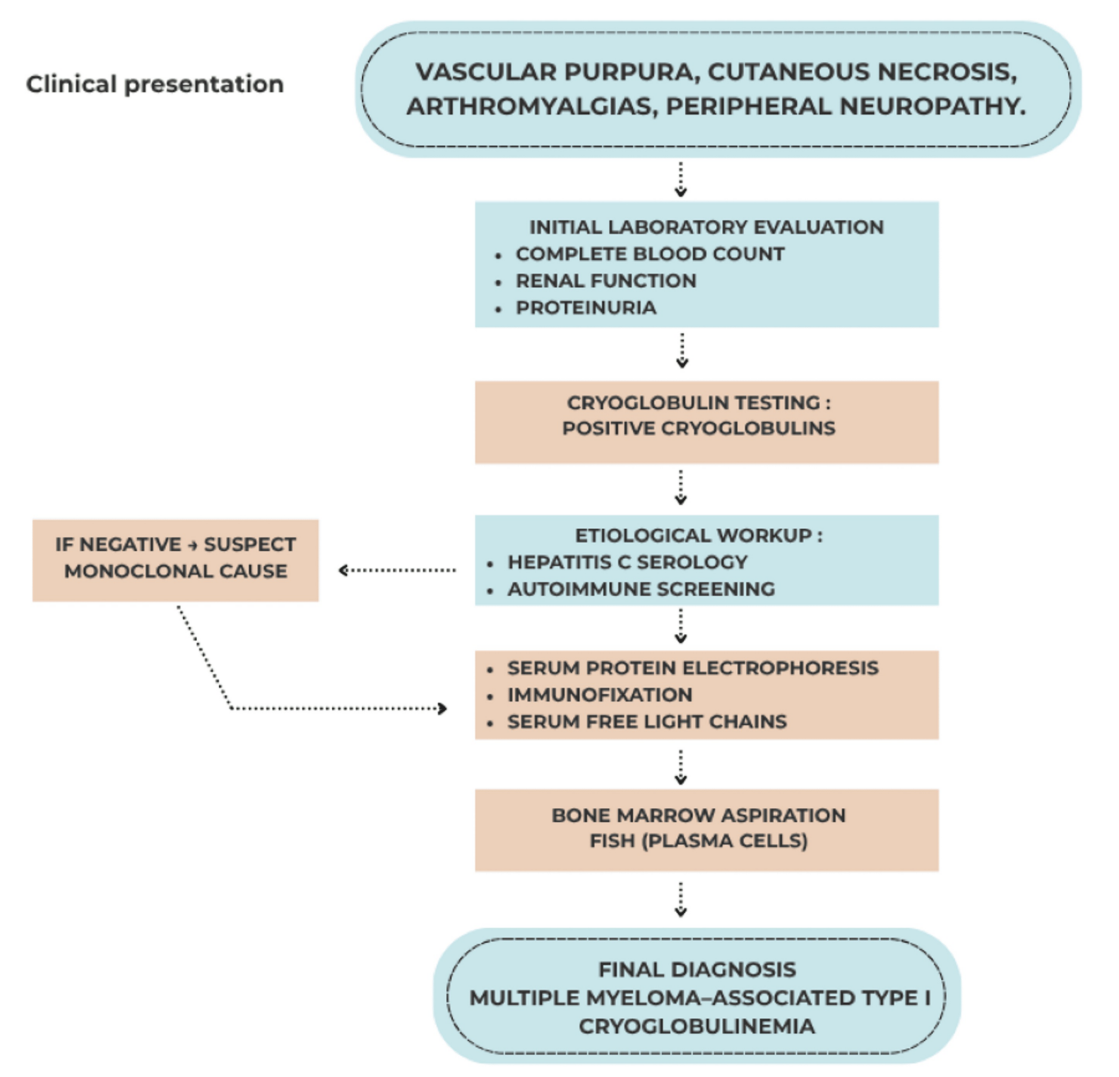
Diagnostic approach to monoclonal gammopathy in the setting of vasculitis.

To better illustrate the diagnostic approach in such cases, we propose a stepwise workflow for the evaluation of suspected monoclonal gammopathy in the setting of vasculitis (Figure 6). This workflow is adapted to our case and may help clinicians in the diagnostic evaluation of similar presentations.

Therapeutic decisions were consistent with current expert-based recommendations, emphasizing management of the underlying clonal disorder as the primary treatment of type I CG. Given the absence of robust randomized data, available guidelines rely largely on expert consensus. In cases secondary to MM, management follows standard MM treatment pathways, with specific adaptations based on organ involvement. Bortezomib-based regimens are recommended as first-line therapy in this case due to their rapid cytoreductive effect and favorable safety profile in patients with renal impairment. This is important in cryoglobulinemic vasculitis with organ involvement, where reduction of the pathogenic monoclonal protein is required. In our patient, significant proteinuria and renal dysfunction supported the initiation of a bortezomib-based regimen. Accordingly, VCD was selected as initial therapy, resulting in rapid clinical improvement and regression of cutaneous lesions [4].

In patients presenting with neuropathy, lenalidomide-based combinations may be favored, whereas autologous stem-cell transplantation remains an option for selected candidates but requires careful assessment of potential cryoglobulin-related organ injury. Plasma-exchange therapy is reserved for symptomatic hyperviscosity or for prevention of IgM flare in Waldenström-associated CG, scenarios not applicable in our case. Supportive measures remain essential to prevent worsening ischemic damage, most importantly, avoidance of cold exposure and attentive skin and limb care [4]. The rapid clinical improvement observed after the initiation of bortezomib in our patient, with marked reduction of pain and early regression of cutaneous lesions, further reinforces the role of prompt clone-directed treatment in MM-associated type I CG.

Dietary management in CG is not well established. Authors have suggested that a low-antigen-content (LAC) diet can reduce immune complex formation, decreasing cryoglobulin levels and improving vascular symptoms such as purpura and vasculitis. These studies often cite mixed CG, but similar nutritional management for reducing immune complexes may also apply to symptomatic type I cases [10].

This report is limited by its single-case design, the rarity of MM-associated type I CG, and its observational nature, which precludes definitive therapeutic conclusions. In addition, the short follow-up does not allow assessment of long-term outcomes.

## Conclusions

This case reinforces the importance of considering MM in patients presenting with type I CG, particularly when ischemic skin lesions or renal involvement occur. Kidney biopsy and comprehensive laboratory evaluation are essential for accurate diagnosis and guiding therapy. The favorable early response to bortezomib observed in our patient highlights the importance of prompt, clone-directed treatment. Given the rarity of MM-associated CG, multicenter registries are needed to better define its epidemiology, clinical course, and optimal management strategies.
